# A Case of Cardiac Arrest Due to Transcatheter Aortic Valve Infolding

**DOI:** 10.7759/cureus.43847

**Published:** 2023-08-21

**Authors:** Fawaz Mohammed, James C Gubitosa, Travis R Huffman, Mohammad Abdul-Waheed, Rahil Rafeedheen

**Affiliations:** 1 Internal Medicine, University of Kentucky College of Medicine, Bowling Green, USA; 2 Cardiology, University of Kentucky College of Medicine, Bowling Green, USA; 3 Cardiology, The Medical Center, Bowling Green, USA; 4 Cardiology, Med Center Health, Bowling Green, USA

**Keywords:** transcatheter aortic valve implantation (tavi), tavr, in-hospital cardiac arrest, heart failure with reduced ejection fraction, bicuspid aortic valve disease, aortic valve insufficiency, interventional cardiologist, tavr complication, structural heart disease intervention, aortic stenosis (as)

## Abstract

Prosthetic valvular infolding during transcatheter aortic valve implantation (TAVI) is an under-recognized yet significant complication that can occur. Here, we describe the case of a 61-year-old male with a history of heart failure with reduced ejection fraction (HFrEF) and low-flow, low-gradient severe aortic valve stenosis of a bicuspid aortic valve who presented to undergo TAVI. During the procedure, repositioning of the valve resulted in prosthetic valvular infolding and resultant severe aortic regurgitation (AR), culminating in cardiac arrest. Swift balloon valvuloplasty corrected the valve geometry and eliminated any AR, allowing hemodynamic recovery and completion of the procedure. Our case and review highlight methods, both angiographic and echocardiographic, to recognize prosthetic valvular infolding the moment it presents, as well as strategies to correct the infolding with minimal detriment to the patient.

## Introduction

Transcatheter aortic valve implantation (TAVI), performed with a self-expandable or balloon-expandable device, is a widely practiced and accepted therapy for patients with severe aortic stenosis. It has been proven to be non-inferior to surgical aortic valve replacement (SAVR) in randomized controlled trials [1]. Frames of self-expanding valves are susceptible to distortion leading to complications. Valvular infolding is a rare, likely under-reported complication seen with these devices with an incidence rate of approximately 3% [2]. Given its rare occurrence, data on this complication are sparse, and it is often missed, leading to paravalvular regurgitation and/or even hemodynamic compromise in severe cases.

Herein, we describe the case of frame infolding of a Medtronic Evolut FX self-expanding bioprosthetic aortic valve (Minneapolis, MN, USA), which was remedied with balloon valvuloplasty post-deployment. Balloon valvuloplasty corrected the valve geometry and eliminated any aortic regurgitation (AR), allowing hemodynamic recovery and completion of the procedure. This case stresses the importance of high-quality imaging and the capacity for rapid intraprocedural decision-making during TAVI.

## Case presentation

**Figure 1:**
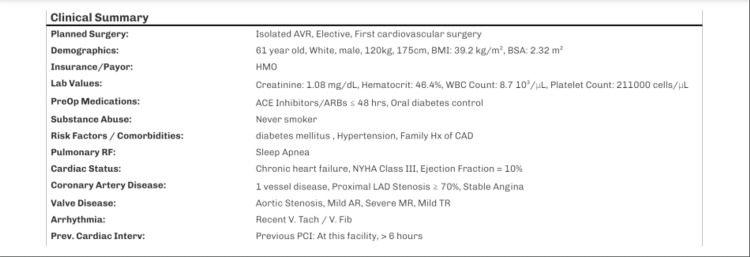
STS score breakdown STS: Society of Thoracic Surgery, AVR: aortic valve replacement, BMI: body mass index, BSA: body surface area, WBC: white blood cell, ACE: angiotensin-converting enzyme, ARBs: angiotensin receptor blockers, CAD: coronary artery disease, NYHA: New York Heart Association, LAD: left anterior descending artery, AR: aortic regurgitation, MR: mitral regurgitation, TR: tricuspid regurgitation, PCI: percutaneous coronary intervention

**Figure 2:**
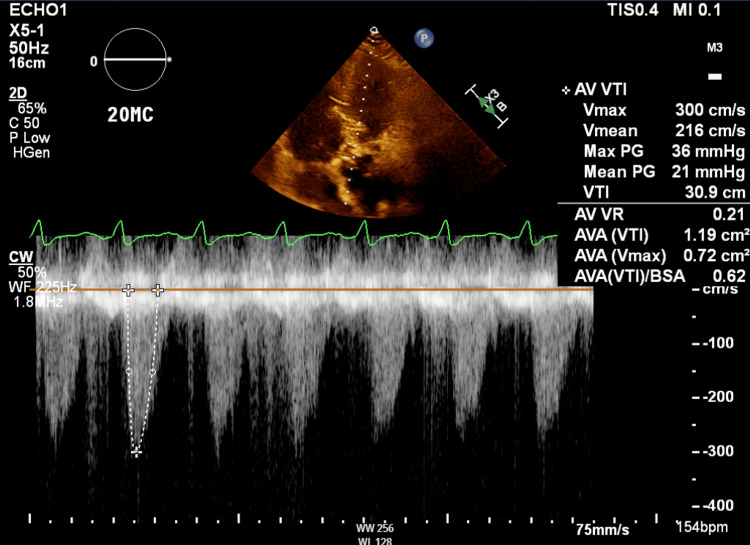
Preprocedural continuous wave Doppler of the aortic valve during dobutamine stress echocardiography at the apical four-chamber view, with findings consistent with severe valvular aortic stenosis

A 61-year-old male with a history of class III obesity, obstructive sleep apnea (OSA), diabetes, hypertension, hyperlipidemia, coronary artery disease (CAD) with prior percutaneous coronary intervention (PCI), heart failure with reduced ejection fraction (HFrEF) recently evaluated to be 10%-15%, ventricular tachycardia status post implantable cardioverter-defibrillator (ICD) placement, and low-flow, low-gradient severe aortic valve stenosis presented to undergo TAVI via right femoral access. The decision was made to pursue TAVI as opposed to surgical aortic valve replacement given his elevated Society of Thoracic Surgery (STS) score indicating a 4.98% risk of operative mortality (Figure 1). Prior to the procedure, he underwent a dobutamine stress echocardiogram, which resulted in a decrease in the calculated aortic valve area (AVA) to 0.72 cm^2^ (Figure 2). Three-dimensional (3D) imaging via transesophageal echocardiography (TEE) was also performed prior to the procedure (Video 1).

**Video 1:** Preprocedural 3D TEE mid-esophageal short-axis view of the aortic valve demonstrating a bicuspid valve architecture 3D: three dimensional, TEE: transesophageal echocardiography

**Figure 3:**
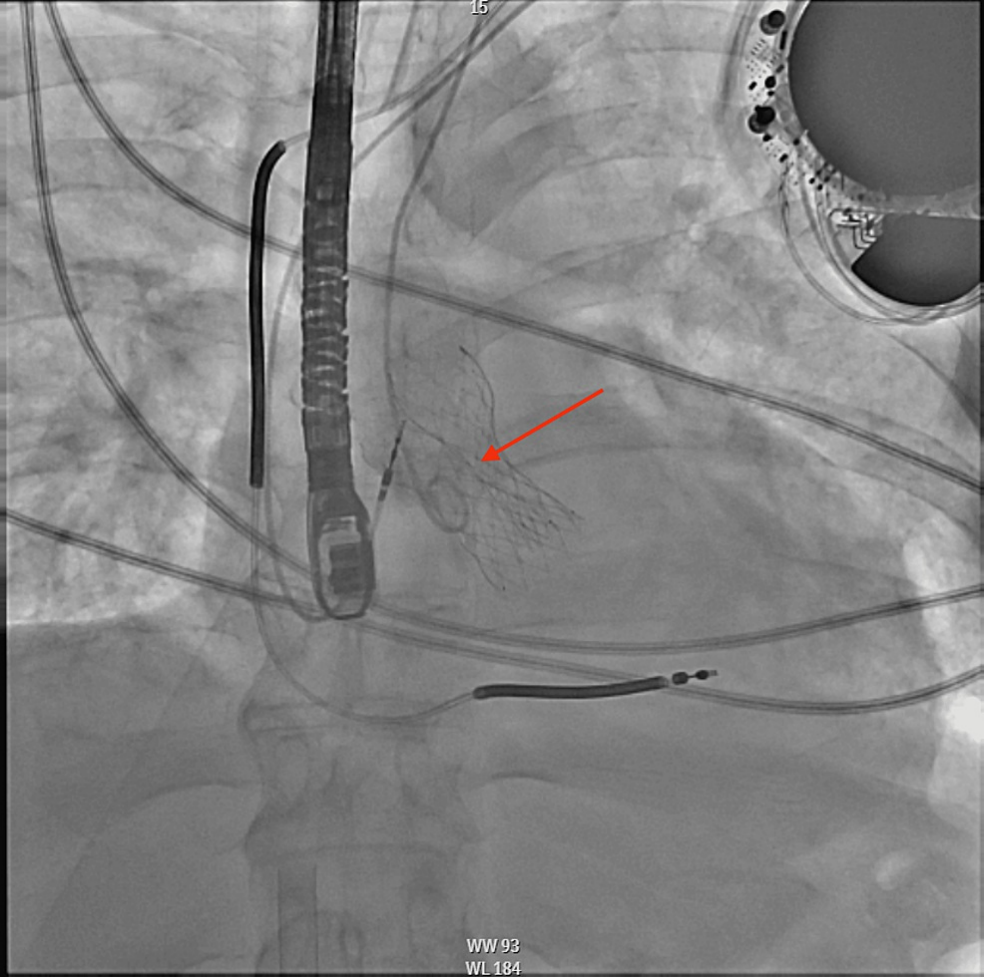
Intraprocedural AP fluoroscopic image of transcatheter aortic valve infolding demonstrating the string sign (red arrow) AP: anteroposterior

**Figure 4:**
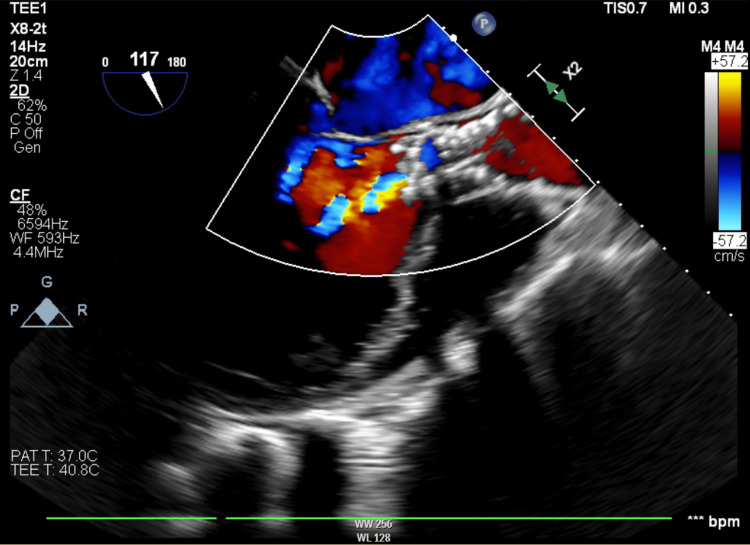
Intraprocedural TEE of the aortic valve with color Doppler, mid-esophageal long-axis view, demonstrating severe prosthetic valvular regurgitation as a result of the infolding TEE: transesophageal echocardiography

**Figure 5:**
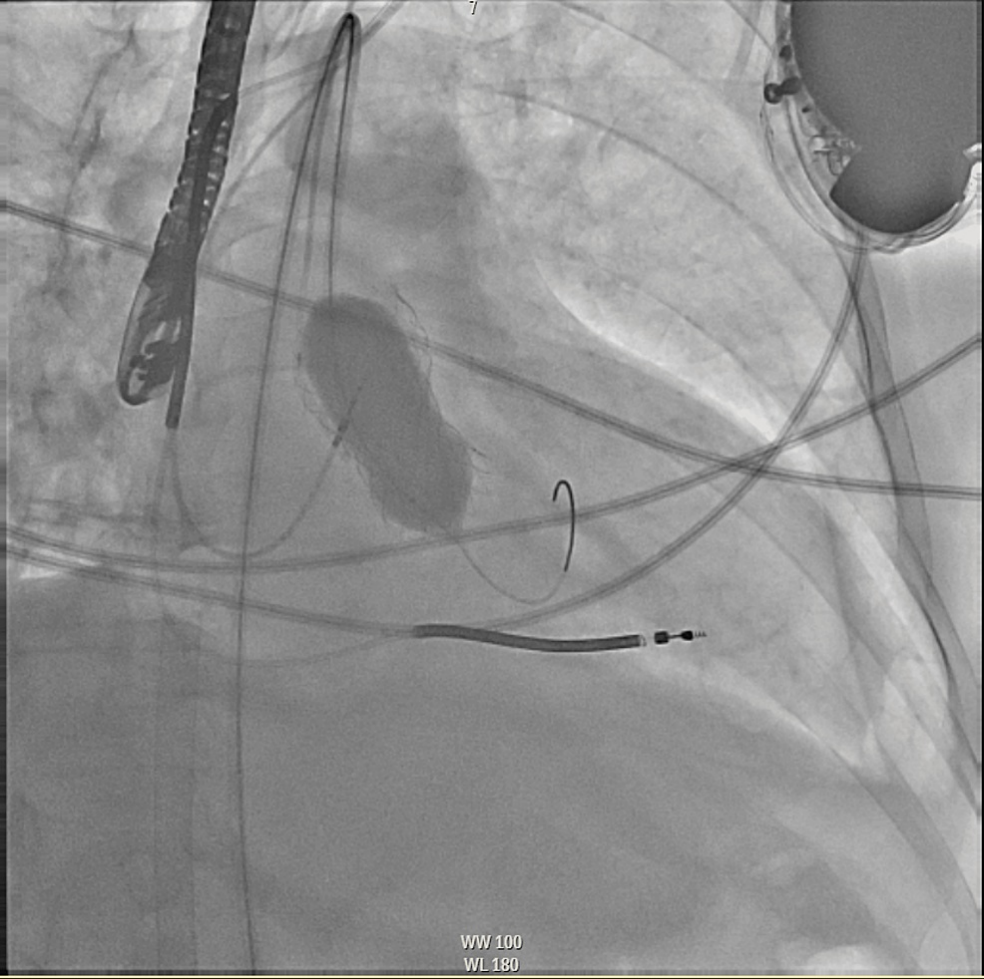
Prosthetic aortic balloon during valvuloplasty

**Figure 6:**
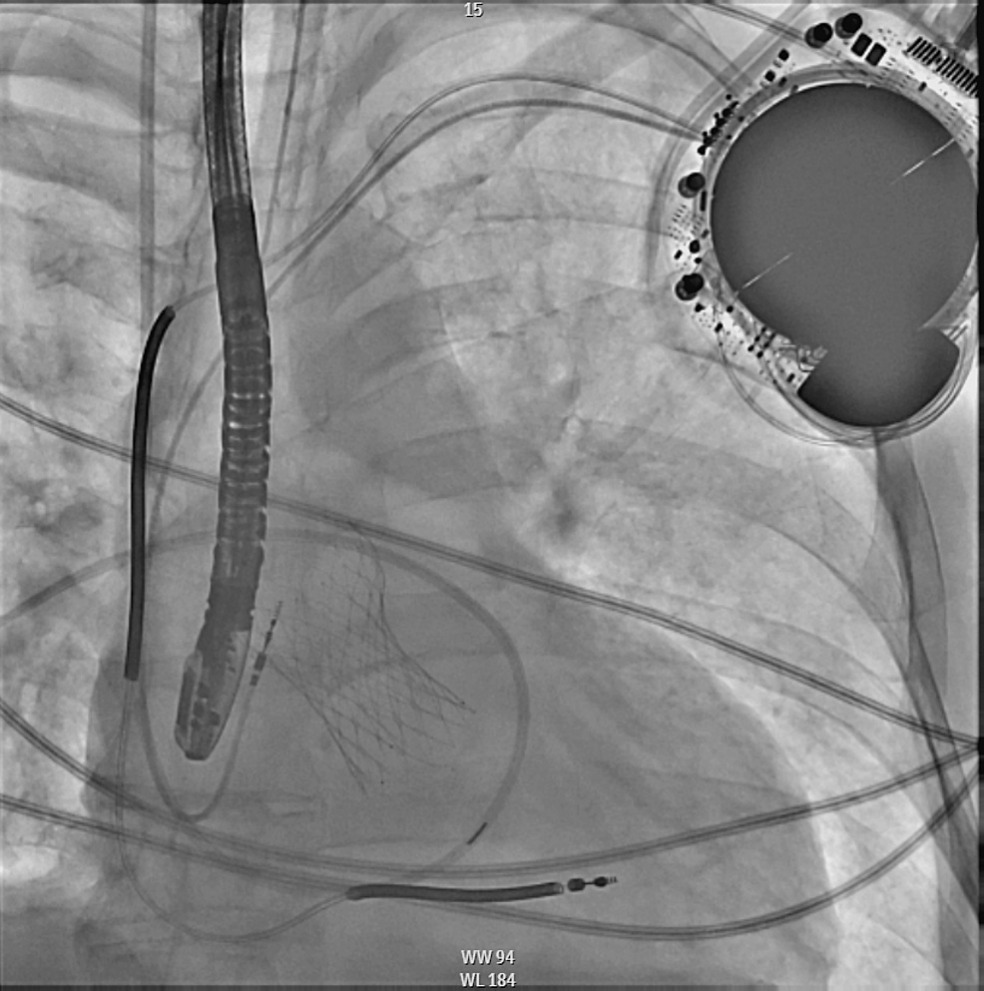
Prosthetic aortic valve post-valvuloplasty with no fluoroscopic evidence of infolding

**Figure 7:**
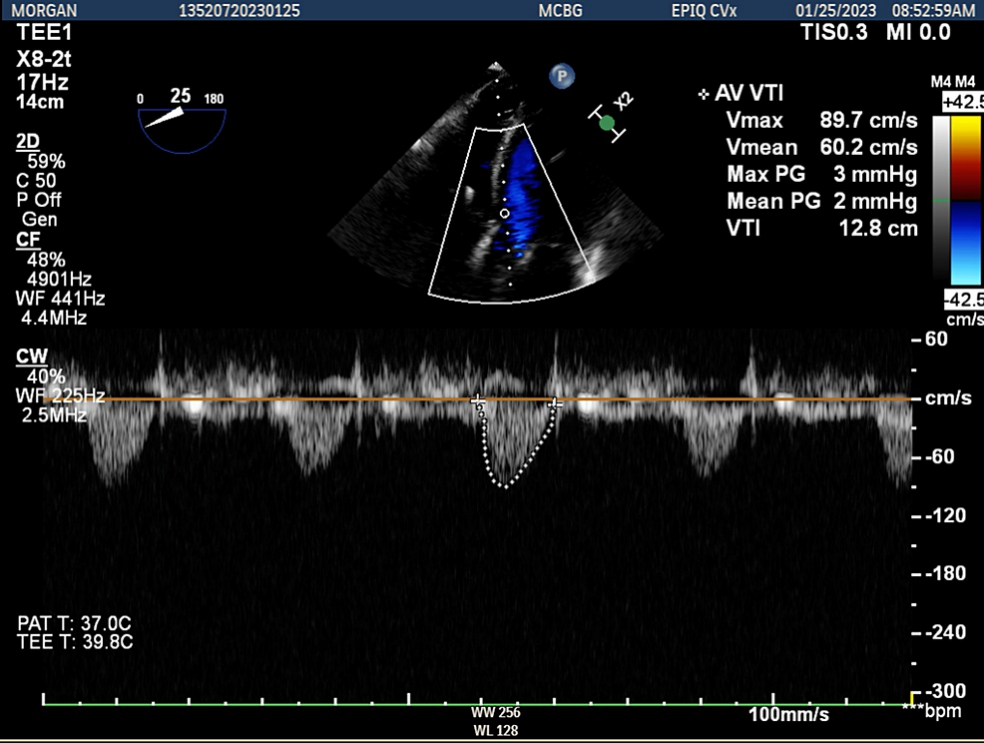
Continuous wave Doppler of the aortic valve post-valvuloplasty

The patient was brought to a hybrid operating room, prepped, and draped. After a time-out, the patient was sedated and intubated by anesthesia. Concomitant TEE was performed during the procedure. A right femoral cutdown was performed. Coronary cusps were delineated via ascending aortography. The valve of choice was a 34-mm Medtronic Evolut FX self-expanding valve (Minneapolis, MN, USA). The prosthetic valve was deployed across the native valve to 80% during left ventricular pacing at 120 bpm when it was observed to be too deep. The valve was recaptured, repositioned, and redeployed to 100%. Repeat valve position was adequate; however, significant valve infolding was noted (Figure 3) with severe prosthetic valvular regurgitation present (Figure 4). The patient became progressively more hypotensive and went into cardiac arrest. Cardiopulmonary resuscitation (CPR) was initiated immediately, and return of spontaneous circulation (ROSC) was obtained within two minutes. The left femoral artery was accessed, and a 24-mm balloon was advanced across the infolded prosthetic valve and post-dilated with rapid pacing (Figure 5). Repeat imaging demonstrated a well-expanded valve (Figure 6) with no significant paravalvular leak, valvular regurgitation, or gradient (Figure 7). The patient was extubated uneventfully later that day and was discharged home the following day in satisfactory condition.

## Discussion

TAVI has become a viable option to manage severe aortic stenosis in patients across the surgical risk spectrum. The commonly used prostheses include either balloon-expandable or self-expandable valves. These designs have been comparable with each other, with each having its advantages and disadvantages. Prosthetic valve infolding is a rare but serious complication encountered in TAVI, ultimately resulting in valve distortion and aortic regurgitation. Infolding can also prolong procedure time, exposing patients to increased procedural risk. Reports have described acute kidney injury and atrioventricular blocks with subsequent need for a permanent pacemaker as a result of valve infolding [3].

Given the rarity and subtlety of infolding, it is often not recognized. Risk factors that have been identified include large-sized prostheses (≥29 mm), severe native valve calcification, recapturing of self-expanding valves, and the presence of a bicuspid aortic valve [4,5]. It is exclusively observed in self-expanding valves. When performing recapture with the larger prosthesis, multiple views should be obtained to closely assess the frame at the time of the second deployment to rule out infolding before fully releasing the valve.

Assessing the valve through fluoroscopic imaging can also help identify this complication. On fluoroscopy, infolding causes the diameter of the prosthesis to appear narrower than expected. The string sign is also observable on fluoroscopy and is described as a vertical line seen along the frame of the valve and is comparable to a matted fish netlike appearance. This phenomenon can be well-observed via the red arrow in Figure 3 with its subsequent resolution in Figure 4. Fluoroscopic evidence of infolding is also well-observed via rotational fluoroscopy. The Pac-man sign, a very subtle D shape seen due to the infolding of the stent frame, may be seen on transesophageal echocardiography (TEE) at the mid-esophageal short-axis view [6].

A dreaded complication of valve infolding is severe aortic regurgitation, and in patients with dilated cardiomyopathy, hemodynamic compromise can result. In an analysis by Ancona et al., severe aortic regurgitation in TAVI resulted in hemodynamic collapse and cardiac arrest in 29% and 12% of cases, respectively. A few cases of death and stroke were also reported [4].

Swift and appropriate resuscitative measures are crucial to tackling hemodynamic instability. Structuralists should anticipate the possibility of infolding with each case and be prepared to supply mechanical or surgical support if necessary. Salvaging and deploying a new device may be needed. If fully deployed, as with our case, valvuloplasty with a suitable balloon should be performed to correct the valvular symmetry and eliminate aortic regurgitation. Although infolding often resolves with balloon post-dilation, stroke is a complication that tends to occur in a subset of patients [7].

Regarding the etiology of our patient’s infolding, we attribute his infolding to both the recapturing of a large 34-mm valve and calcification present on the fused leaflets of his native bicuspid valve, which further adulterates native valve geometry. His cardiac arrest was likely then secondary to the resultant severe AR further impacting his already severely reduced left ventricular systolic function.

## Conclusions

Prosthetic valvular infolding during TAVI is a rare and subtle yet devastating complication that merits further recognition. Data is limited, and further studies are needed to recognize factors that may predispose patients to this complication. As the indications for TAVI continue to expand and the procedure begins to become more prevalent, the incidence of valvular infolding would be expected to increase. Vigilance will be needed on the part of the structuralist and echocardiographer to facilitate its early identification and rapid rectification in order to best mitigate patient morbidity and mortality.
